# Optimising local institutional role for sustainable community preparedness in Krinjing: The slope of Merapi Volcano

**DOI:** 10.4102/jamba.v18i1.2142

**Published:** 2026-08-14

**Authors:** Dewi L. Setyowati, Syamsul Bachri, Andi I. Benardi, Bayu Wijayanto, Sriyono Sriyono, Atta-ur Rahman, Fajar Wulandari, Jaka P. Rahmajati

**Affiliations:** 1 Department of Geography, Faculty of Social and Political Science, Universitas Negeri Semarang, Semarang, Indonesia; 2 Department of Geography, Faculty of Social Science, Universitas Negeri Malang, Malang, Indonesia; 3 Department of Geography, Faculty of Social Science, Universitas Negeri Padang, Padang, Indonesia; 4 Department of Geography and Geomatics, University of Peshawar, Peshawar, Pakistan

**Keywords:** community resilience, disaster management, Merapi Volcano, institutional performance, sustainable community, disaster risk reduction

## Abstract

The effectiveness of local institutions in disaster-prone areas is often assessed with a bias towards administrative aspects or social solidarity alone, without in-depth operational evaluation. This study audits the internal performance of the Disaster Risk Reduction Organisation or Organisasi Pengurangan Risiko Bencana (OPRB) in Krinjing Village, Magelang, facing the threat of Merapi Volcano’s eruption. Using a quantitative descriptive approach with purposive sampling, data were collected through a closed-ended questionnaire administered to 12 key structural informants, followed by in-depth interviews to contextualise the findings. The results showed a significant difference: the OPRB demonstrated superior tactical capacity in the emergency response phase, driven by social capital and logistical independence. However, this advantage is inversely proportional to the pre-disaster phase, marked by top-down planning and minimal participation. In the post-disaster phase, the OPRB showed the lowest performance in monitoring and evaluation, breaking the organisational learning cycle. These findings indicate that the OPRB does not currently function as an adaptive resilience organisation. To transform this capacity, the study formulates three institutional engineering strategies: institutionalising disaster management in the RPJMDes document, formalising local wisdom and decentralising authority. These approaches are expected to close the managerial gap and achieve sustainable community resilience on Merapi’s slopes.

**Contribution:**

This research fills a practical and theoretical gap by demonstrating how the dominance of top-down approaches in local disaster management stifles grassroots resilience, despite strong informal social capital during emergencies. Its primary contribution is a phase-specific evaluation and a strategic transformation model to institutionalise resilience, directly supporting the journal’s scope of disaster risk reduction and resilience.

## Introduction

Situated within the Pacific Ring of Fire, Indonesia faces an extreme level of volcanic vulnerability. Merapi Volcano stands as one of Indonesia’s most active volcanoes, characterised by a short eruption cycle (Gertisser et al. 2023). Over the past 16 years, at least seven significant eruption periods have disrupted the activities of residents on the southwest slopes (Aji et al. 2021; Gertisser et al. 2023). History records that the explosive eruption of Merapi in 2010 claimed more than 300 lives and displaced 300 000 residents (Benardi et al. 2023).

However, the threat does not always manifest on a catastrophic scale. In 2020, Merapi demonstrated its volatility again through a series of phreatic eruptions. Although categorised as relatively safe with limited impact, these events still triggered self-evacuation among residents because of situational uncertainty and past trauma (Nugraha et al. 2021). This dynamic confirms that Merapi’s hazard character is highly fluctuating, demanding community preparedness that is not only responsive to significant eruptions but also adaptive to sudden phreatic threats. The significant variability of these threats indicates that disaster risk in this region demands robust preparedness management (Handoyo et al. 2024; Wulandari et al. 2023).

Krinjing Village in Dukun District, Magelang Regency, stands at the forefront of this risk. Situated within the Disaster-Prone Area or *Kawasan Rawan Bencana* (KRB) III, the village faces direct threats from pyroclastic flows and incandescent lava, as well as rain-triggered lahar floods (Benardi et al. 2023). However, this high physical risk is inversely proportional to the existing social capacity. Preliminary data show a troubling reality: about 40% of residents on Merapi’s slopes lack a full understanding of evacuation procedures and have never taken part in emergency drills (Widati, Sakina & Adiwirahayu 2021). This indicates a sharp disparity between high-risk exposure and low disaster resilience. To bridge this gap, a community-based approach through the Disaster Risk Reduction Organisation/*Organisasi Pengurangan Risiko Bencana* (OPRB) is crucial (Benardi et al. 2023).

The OPRB is a local institution established under the Disaster Resilient Village (*Desa Tangguh Bencana*) programme of the National Agency for Disaster Management. Comprising various societal elements, ranging from village officials to vulnerable groups, the OPRB is mandated to function as a tactical command unit at the grassroot level. Ideally, this organisation serves as a mediator, an agent of action and a primary coordinator across all phases of a disaster, from mitigation and emergency response to recovery (Rasidi et al. 2024). However, the actual effectiveness of the OPRB is frequently assumed to be functioning well without in-depth empirical evaluation (Rasidi et al. 2024).

A literature review reveals that three perspectival biases have thus far constrained OPRB performance evaluations. Firstly, administrative bias, where studies such as Fajria, Putera and Ariany (2023) and Mahendra, Pratomo and Paripurno (2022) tend to assess OPRB success solely on the basis of document completeness and institutional legality. Secondly, socio-cultural bias, as seen in studies by Fauzie and Sariffuddin (2017) and Mutiarni, Nakamura and Bhattacharya (2022), which focus on social solidarity, constructs a narrative that the spirit of *gotong royong* [cooperation] automatically guarantees OPRB performance without considering comprehensive disaster management aspects. Thirdly, technocratic bias, as in the research by Vasileiou, Barnett and Fraser (2022), which overemphasises the integration of early warning systems and assumes that the OPRB will function effectively solely on the basis of accurate risk information.

These three perspectives leave an empirical gap; there is yet to be a study that critically audits the OPRB’s internal institutional performance, disaggregated by each critical disaster phase. The lack of such an assessment risks fostering a false perception of resilience, whereby a village is considered resilient from an administrative and social perspective yet continues to face operational vulnerabilities.

Theoretically, this study is anchored in three frameworks. The absorptive adaptive transformative (AAT) resilience framework (Béné et al. 2014) provides an evaluative benchmark, mapping absorptive capacity to emergency response, adaptive capacity to risk reduction and transformative capacity to post-disaster recovery, corresponding directly to the three phases examined here. Institutional quality is further adopted as a measurable dimension of adaptive capacity (Khadka 2024), distinguishing formal compliance from substantive operational performance. Finally, the community-based versus community-led governance continuum (Paripurno et al. 2022) establishes the normative direction for the proposed institutional strategies, positioning the OPRB’s transformation as a shift from administrative implementation towards self-organising community resilience.

This study aims to fill this void by evaluating the OPRB’s role in Krinjing Village. The evaluation is conducted comprehensively across all stages of disaster management, from disaster risk reduction and emergency response to rehabilitation. This is essential to uncover the extent of this local institution’s preparedness to face the real threat posed by Merapi Volcano.

## Research methods and design

### Study site

**FIGURE 1:**
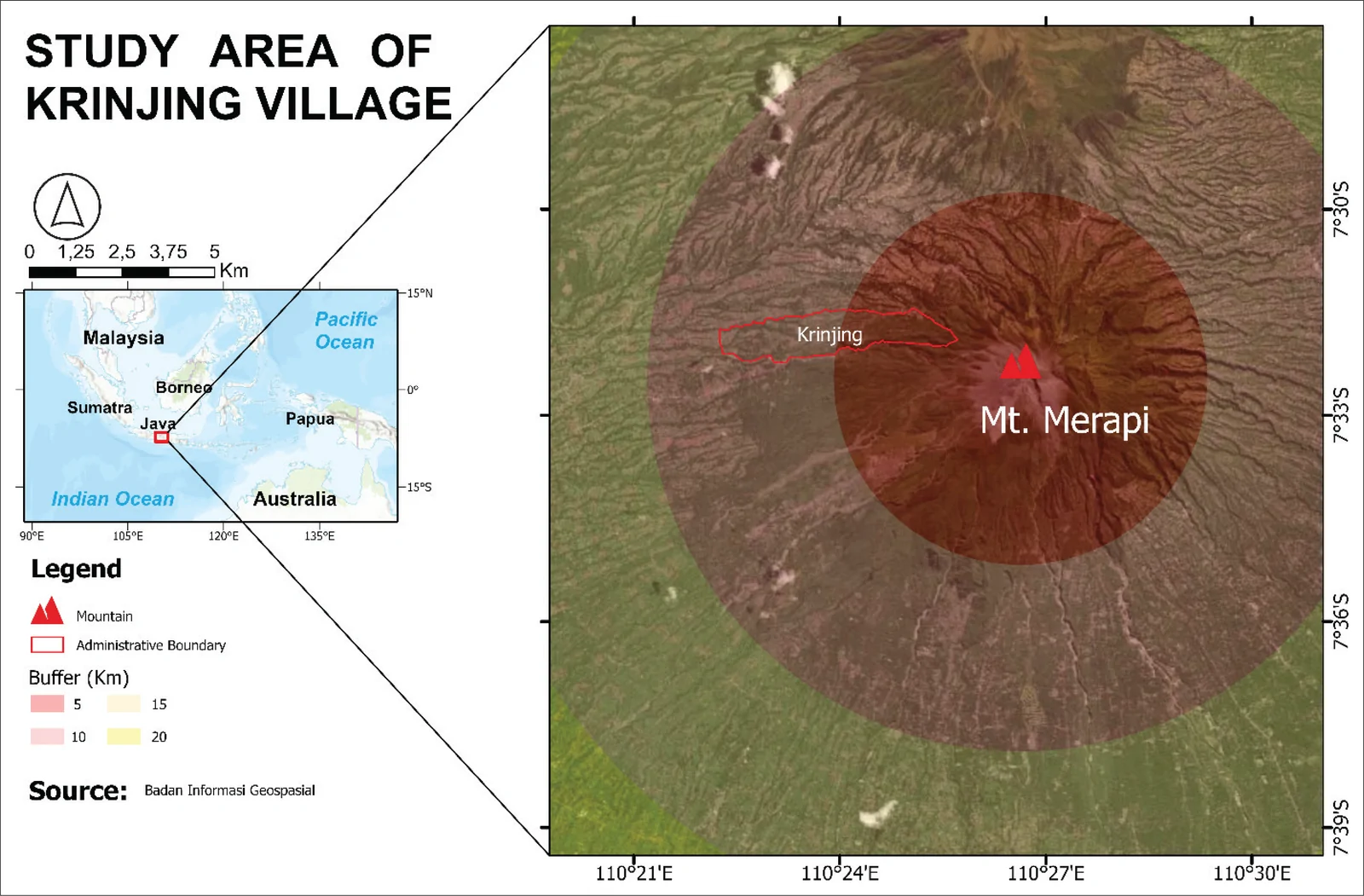
Study area.

Krinjing Village covers an area of 613.15 ha and is situated in Dukun District, Magelang Regency, Central Java Province. Geographically, Krinjing Village is located in the southwest of Merapi Volcano and lies within a 5 km – 10 km radius of the summit (Figure 1). Its proximity to the summit implies a high level of vulnerability to potential eruptions and other volcanic activity (Adjie & Benardi 2026).

Given its proximity to the top of Merapi, Krinjing Village faces physical and social vulnerability. Physically, this vulnerability is exacerbated by the region’s morphology, which is dominated by steep slopes and river channels that originate at the summit of Merapi Volcano. Consequently, this increases the likelihood that residential areas and agricultural lands will be impacted. Meanwhile, the village’s population, as of 2024, has reached 2289 inhabitants. Of this total, at least 38% belong to vulnerable groups, comprising children and the elderly (Krinjing Village Government 2025; Magelang Regency Population Service 2025). This significant figure underscores the need for community-based disaster management and preparedness.

### Data collection

Data were collected through four primary methods (Table 1). Firstly, we used adapted questionnaires to ensure each indicator was representative. Secondly, non-participant observations were conducted to assess the physical environment and residential settlements. Thirdly, in-depth interviews with OPRB administrators provided more profound insights into their local disaster management experiences. Fourthly, document reviews, including hazard maps, evacuation standard operating procedures (SOPs) and demographic data, were used to verify findings and support spatial analysis.

**TABLE 1:** Data types and sources.

Data	Type	Source
The role of OPRB	Primary	Questionnaire
Monograph and population	Secondary	Office of the Head of Krinjing Village and the Magelang Regency Population Office
Structure of Village Disaster Management Institution	Secondary	Krinjing Village Head Office
Map of disaster-prone areas	Secondary	Geological Disaster Technology Research and Development Institute

OPRB, Organisasi Pengurangan Risiko Bencana.

### Population and sampling

The sample of 12 respondents represents 50% of the total OPRB population, a proportion that meets the threshold for an institutional audit, which aims to assess its analytical objectives, which are descriptive rather than inferential (Etikan, Musa & Alkassim 2016). Generalisation beyond Krinjing Village is not the goal; rather, this study seeks an in-depth institutional diagnosis of a limited organisational unit. The exclusion of broader community members as primary respondents was a deliberate methodological decision, as this study evaluates institutional performance from the perspective of those who operate it. To mitigate self-report bias, quantitative scores were systematically triangulated with semi-structured interviews and document reviews, allowing for cross-validation with administrators’ perceptions of observable organisational practices. Inclusion criteria required respondents to be permanent residents of the 2010 and/or 2020 eruption areas, aged 18 years or older, fluent in Indonesian, and participating voluntarily.

### Instrumentation and variable

The research instrument used consisted of a closed-ended questionnaire containing 15 questions, adapted from three variables and seven indicators, designed to quantitatively measure the effectiveness of the OPRB’s role. In-depth interviews were conducted using a semi-structured interview guide to qualitatively explore the socio-cultural context underlying these scores. Table 2 outlines the variables, indicators and corresponding question numbers within the research instrument.

**TABLE 2:** Audit variables, indicators and focus.

Variable	Indicator	Inquiry number	Audit focus
Implementation of disaster risk reduction	Prevention of sampling Mitigation Preparedness	1–5	Preventive measures Planning participation Capacity building commitment Disaster education Logistics readiness and evacuation routes
Implementation of disaster emergency handling	Rescue and evacuation Fulfilment of basic needs	6–10	Evacuation coordination Information management and early warning Evacuation strategy for victims Public kitchen management and logistics Emergency health care
Post-disaster recovery	Rehabilitation Reconstruction	11–15	Social psychological rehabilitation Monitoring and evaluation mechanism Post-disaster public service improvement Post-disaster community participation Physical and non-physical development

### Validity test

Validity was assessed using Pearson’s product-moment correlation, a widely established method for evaluating questionnaire instruments (Taherdoost 2016). Each item’s calculated *r*-value was compared against the critical *r*-table value of 0.361 (*n* = 30, α = 0.05); items with r-count below this threshold were deemed invalid and eliminated. Five items were eliminated for falling below the validity threshold, with *r*-count values of 0.152, 0.343, 0.182, 0.275 and 0.290, respectively. It is important to note that validity testing eliminated specific questionnaire items, not the indicators they were intended to measure. When multiple items measured the same indicator, eliminating one item did not remove the indicator, as it was captured by other valid items. The eliminated items originally addressed two items within the disaster risk reduction phase (preventive measures and disaster education), one item within the emergency response phase (information management and early warning) and two items within the post-disaster recovery phase (monitoring and evaluation mechanism and physical and non-physical development). All of these indicators remain represented in the final instrument through other valid items, as presented in Table 2. The 15 retained items are distributed equally across the three disaster management phases (five items each), ensuring adequate construct coverage across all dimensions evaluated in this study.

### Data analysis

To facilitate quantitative analysis of the OPRB’s role, the questionnaire utilised a 5-point Likert scale with five response alternatives, as detailed in Table 3. Subsequently, for the frequency distribution analysis, the total scores from the 15 questions were calculated to obtain a cumulative score for each respondent (Min = 15; Max = 75). These scores were then classified into five interval classes with a range of 12, as presented in Table 3.

**TABLE 3:** Likert scale criteria for the role of Organisasi Pengurangan Risiko Bencana.

Role classification	Score	Score range
Very significant	5	63 < 75
Significant	4	51 < 63
Moderately significant	3	39 < 51
Low significance	2	27 < 39
Insignificant	1	15 < 27

Meanwhile, qualitative data from in-depth interviews and document reviews were analysed using data source triangulation, in which information from different informants, including community figures, volunteers and village officials, was compared and cross-verified to ensure credibility (Carter et al. 2014). These triangulated insights were used to corroborate and provide deeper contextual explanations for the quantitative Likert scores, particularly regarding socio-cultural dynamics and the integration of local wisdom. To examine whether performance differences across phases were statistically significant, a Friedman test was additionally employed. This nonparametric test was selected as appropriate given the ordinal nature of Likert-scale data and the small sample size (*n* = 12), serving as a repeated-measures alternative to analysis of variance (ANOVA) that does not assume normality (Ross 2009).

### Ethical considerations

Ethical clearance to conduct this study was obtained from the Institute for Research and Community Engagement (Ref. No. 24.2.21/UN32.20/PB/2026).

## Results

### General overview of Organisasi Pengurangan Risiko Bencana performance

Table 4 presents a general overview of the OPRB’s performance in Krinjing Village, which was overall assessed as moderately significant with an average score of 45.

**TABLE 4:** General overview of Organisasi Pengurangan Risiko Bencana performance.

Variable	*N* (sample)	Min. score	Max. score	Mean total score	Performance category
OPRB	12	40	50	45	Moderately significant

OPRB, Organisasi Pengurangan Risiko Bencana; min., minimum; max., maximum.

While the overall assessment falls within the moderately significant category, this average figure obscures a significant performance disparity across different disaster phases. This categorisation is essentially a composite of robust performance during the emergency response phase, by suboptimal performance in the planning and recovery phases, as discussed in detail in the subsequent section. The Friedman test confirms that this performance disparity across phases is statistically significant (χ^2^[2] = 19.54, *p* < 0.001), indicating that the observed differences in institutional capacity between the disaster risk reduction, emergency response and post-disaster recovery phases are not attributable to random variation but reflect genuine structural gaps in OPRB’s organisational performance.

### Implementation of disaster risk reduction

The OPRB’s role in disaster risk reduction activities in Krinjing Village shows an imbalance in contributions during the disaster risk reduction phase, as observed in Table 5.

**TABLE 5:** The role of Organisasi Pengurangan Risiko Bencana in disaster risk reduction.

Indicator	Mean score (1–5)	Performance category
Preventive measures	2.92	Moderately significant
Planning participation	2.5	Low significant
Capacity building commitment	4.08	Significant
Disaster education	4.17	Significant
Logistics readiness and evacuation routes	4.33	Significant
Average	3.6	Moderately significant

Pre-disaster evaluation reveals a striking gap between OPRB’s technical capacity and social legitimacy (Table 5). Planning participation recorded the lowest score (2.58), indicating a persistent top-down approach in which OPRB functions merely as an administrative unit, prioritising bureaucratic compliance over citizen engagement. When village elites dominate mitigation planning, residents perceive disaster management as solely the government’s responsibility (Monsalve, Valladares & Díaz 2024). This lack of participation undermines social trust, as Zhang et al. (2019) and Fan (2015) caution, and neglect of the community during the planning process markedly heightens the likelihood of dissatisfaction and horizontal conflicts during the reconstruction phase.

This issue is compounded by the low Prevention Efforts score (2.92), reflecting an unresolved epistemological clash. Merapi residents remain deeply attached to local wisdom systems – personified by the Locksmith [*Juru Kunci*] (e.g. the late Mbah Maridjan) – and often trust natural signs over formal instructions (Permana 2020b).

The data confirm OPRB’s failure to translate modern disaster science into acceptable cultural narratives. While integrating local knowledge is crucial to protect vulnerable groups, aligning formal SOPs with local culture is frequently hindered by logistical constraints and stakeholder fatigue (Rozi, Ritonga & Januar 2021; Vasileiou et al. 2022). In Krinjing Village, this unresolved challenge impedes grassroots prevention efforts.

Conversely, OPRB excels operationally. Compelling scores in Logistics Readiness (4.33), Disaster Education (4.17) and Capacity Building Commitment (4.08) demonstrate its successful transformation into an effective tactical unit capable of mobilising resources, preparing evacuation routes and rapidly disseminating early warnings. However, this creates a competency paradox: Krinjing Village is logistically disaster-ready but socio-politically fragile. A dominant command-and-control approach often sidelines residents’ critical awareness. Imperiale and Vanclay (2019) argue that highly technical disaster management lacks empowerment, risks facilitating disaster capitalism and fosters dependency on external aid. Consequently, resilience remains contingent on OPRB administrators rather than the community’s collective consciousness.

### Implementation of disaster emergency response

Transitioning to the emergency response phase, the evaluation shows a significant improvement in performance over the previous phase. The detailed assessment of each emergency response indicator is presented in Table 6.

**TABLE 6:** The role of Organisasi Pengurangan Risiko Bencana in disaster emergency response.

Indicator	Mean score (1–5)	Performance category
Evacuation coordination	3.75	Moderately significant
Information management and early warning	4.08	Significant
Evacuation strategy for victims	4.08	Significant
Public kitchen management and logistics	4.58	Very significant
Emergency health care	4.33	Significant
Average	4.16	Significant

Emergency response performance significantly exceeded the risk-reduction phase (Table 6). The highest score in Communal Kitchen and Logistics Management (4.58) highlights Krinjing Village’s logistical self-sufficiency, driven by local initiative rather than passive reliance on external aid. This confirms the effectiveness of pre-disaster preparations, underpinned by substantial social capital. As Mutiarni et al. (2022) note, women’s roles and *gotong royong* [cooperation] are crucial for securing emergency logistics on Merapi’s slopes, often enabling faster responses than government interventions (Imperiale & Vanclay 2019). However, despite this substantial grassroots capital, long-term logistical sustainment still necessitates local government support.

**FIGURE 2:**
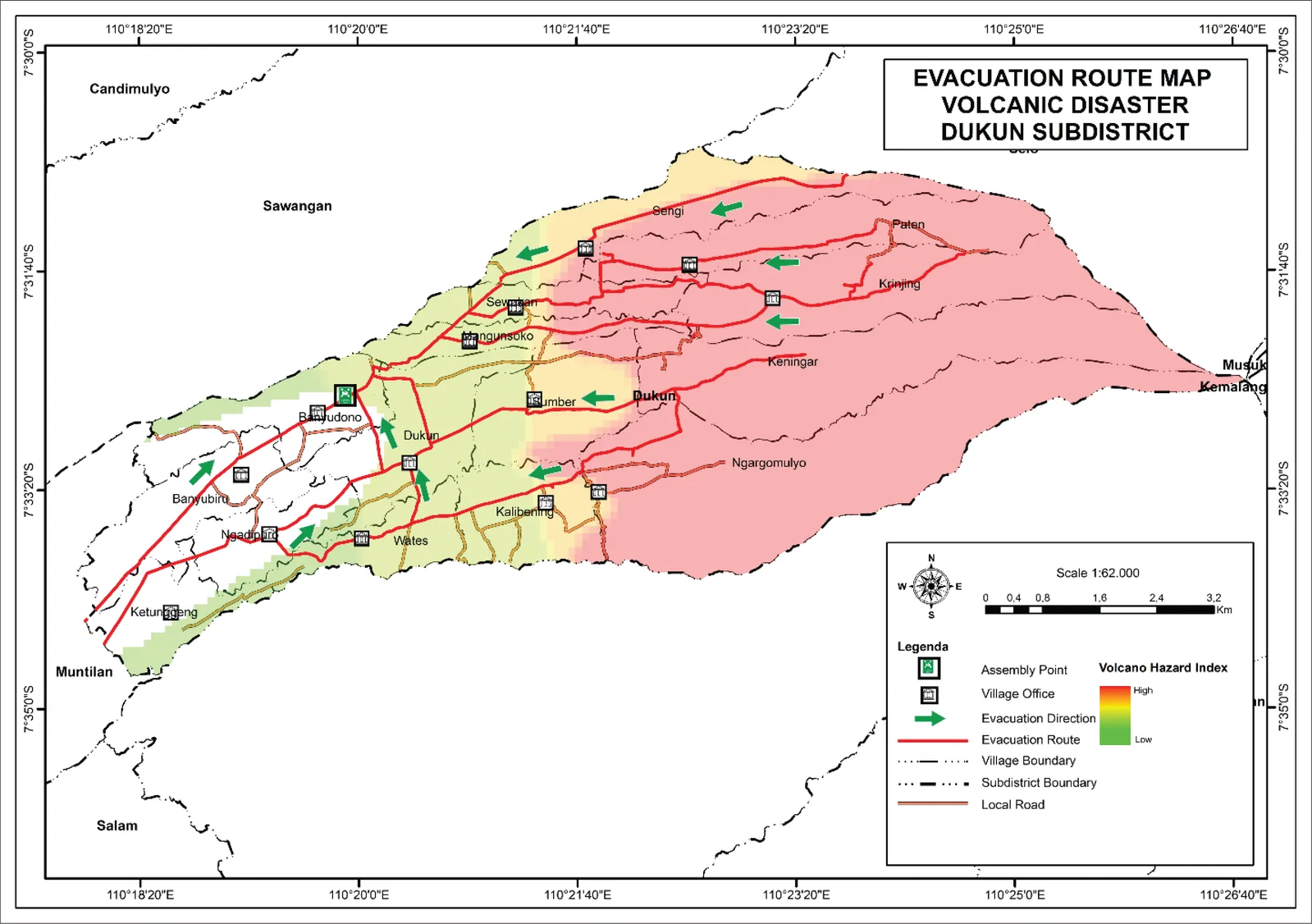
Evacuation route map.

Organisasi Pengurangan Risiko Bencana also exhibited solid performance in Evacuation Strategy and Information Centre indicators (both 4.08), reflecting successful information dissemination and activation of pre-prepared routes (Figure 2), supported by local Geological Agency and Badan Nasional Penanggulangan Bencana (BNPB) observation posts. Nevertheless, cultural challenges persist. Relying heavily on local knowledge, residents often favour informal channels and await directives from traditional leaders or the Locksmith [*Juru Kunci*] (Permana 2020a, 2020b), preventing OPRB from serving as the sole information node. This duality risks triggering ambiguity and impeding evacuation if threat assessments diverge (Mei et al. 2013), a direct residue of excluding local knowledge from formal planning protocols.

Additionally, the most significant shortcomings in community leadership, rooted in the trust cultural value, can undermine their sustainability and activity if local mobilisers remain inactive and no new active individual leaders are regenerated (Ruslanjari et al. 2024). Conversely, residents’ practical experience naturally accelerates the speed of tactical responses (Vasileiou et al. 2022).

For Emergency Health Handling (4.33), OPRB acted as a vital frontline, utilising *gotong royong* [cooperation] to provide first aid to vulnerable groups, although limited capacity eventually required external medical support. Refugee coordination scored comparatively lower (3.75), which is expected given the chaotic onset of the disaster. In this context, OPRB’s command structure emerged as a primary strength; unlike the planning phase, which necessitates deliberation, emergency response demands rapid, decisive tactical decision-making, which this command approach facilitates (Imperiale & Vanclay 2019).

### Post-disaster recovery

In contrast to the role observed during the emergency response phase, the evaluation results for the post-disaster recovery phase indicate a drastic decline in performance, with an average score of 2.71, classified as low significance, as shown in Table 7.

**TABLE 7:** The role of Organisasi Pengurangan Risiko Bencana in post-disaster recovery.

Indicator	Mean score (1–5)	Performance category
Social psychological rehabilitation	4.08	Significant
Monitoring and evaluation mechanism	1.83	Insignificant
Post-disaster public service improvement	3.33	Moderately significant
Post-disaster community participation	2.17	Low significant
Physical and non-physical development	2.17	Low significant
Average	2.71	Low significant

Following the emergency response, OPRB’s operational capacity declined significantly (Table 7). However, Social-Psychological Rehabilitation remained significant (4.08), demonstrating that residents’ social capital and spirit of unity serve as a crucial cultural coping mechanism for holistic recovery (Oflazoğlu & Dora 2024). Women play a pivotal role in this mechanism through welfare, child education and the management of social insurance schemes when formal aid falls short (Mutiarni et al. 2022). This underscores social capital as Krinjing Village’s primary post-disaster strength.

Conversely, Monitoring and Evaluation (M&E) received the lowest score (1.83). This insignificant role represents a critical failure to identify deficiencies across all disaster phases, causing OPRB to miss valuable lessons for rectifying risk management strategies. Without robust M&E, resident participation risks further diminishment, inevitably causing subsequent risk-reduction efforts to revert to an ineffective top-down approach (Imperiale & Vanclay 2019). This evaluative failure directly impedes Post-Disaster Public Service Improvement (3.33); without a solid database, OPRB merely maintains routines rather than achieving accurate adaptation, causing community resilience to stagnate (Mei et al. 2013; Shneiderman et al. 2021).

The OPRB’s failure in M&E is also reflected in its role in Post-Disaster Public Service Improvement, which was assessed as merely moderately significant (3.33). This finding indicates that the OPRB’s efforts to adjust SOPs and enhance post-disaster service capacity have only reached the level of maintaining routines, rather than achieving adaptation. Furthermore, this reveals an anomaly in which the OPRB attempts to implement post-disaster public services without a solid database underpinning the M&E process. This situation results in a lack of substantial improvements, consequently causing community resilience to stagnate rather than evolve in the face of disasters (Mei et al. 2013; Shneiderman et al. 2021).

Furthermore, Post-Disaster Community Participation scored a low significance (2.17), highlighting OPRB’s failure to decentralise authority and establish hamlet-level preparedness teams. This centralisation undermines grassroots-level handling, stalling the necessary shift from community-based to community-led disaster governance (Paripurno et al. 2022). Similarly, the reconstruction process (2.17) remains hindered. The OPRB’s efforts were primarily limited to basic damage assessments and the construction of inadequate temporary shelters, revealing a focus on a purely physical reconstruction paradigm. Ironically, despite the residents’ rich social capital, OPRB’s inability to facilitate systemic socio-economic recovery leaves the community vulnerable and dependent on external post-disaster aid (Rahman & Van den Broeck 2025).

## Discussion

### Strategy to enhance the role of Organisasi Pengurangan Risiko Bencana

The comprehensive evaluation of the OPRB’s role in disaster management in Krinjing Village reveals significant performance disparities across each phase. These findings necessitate institutional re-engineering to transform the OPRB into a sustainably resilient organisation. This transformation must be pursued through three strategic approaches: policy institutionalisation, the integration of local wisdom and participatory decentralisation.

**FIGURE 3:**
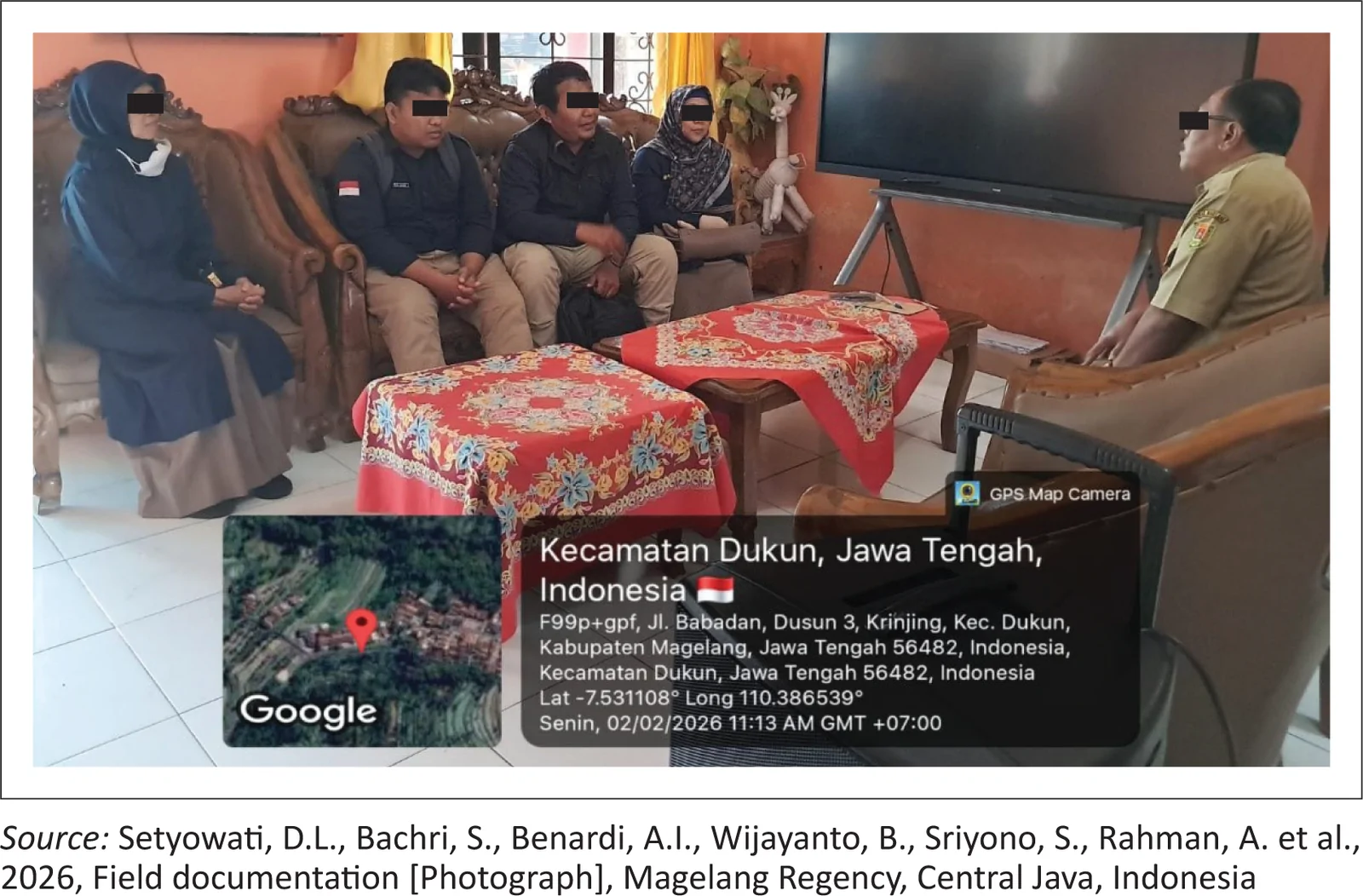
Interview session between the research team and the Head of Krinjing Village during field data collection, Dukun Subdistrict, Magelang Regency (02 February 2026).

The first approach involves the institutionalisation of disaster management through integration into the Village Medium-Term Development Plan or *Rencana Pembangunan Jangka Menengah Desa* (RPJMDes). This strategy is essential to address the top-down approach that dominates the disaster risk reduction phase and the M&E function’s weakness during the recovery phase. This budgetary challenge was corroborated through an interview with the village head (Figure 3), who indicated that village funds are frequently prioritised for other sectors, such as infrastructure development, leaving disaster-related activities with limited budgetary priority.

Consequently, preparedness programmes, including training, simulations and equipment maintenance, often receive insufficient and inconsistent funding, underscoring the need to secure disaster management as a fixed budgetary commitment. A concrete step is to integrate the entire disaster risk management cycle into the RPJMDes document. This integration is crucial for accommodating vital activities that have been neglected, specifically by allocating the village fund to establish a citizen participatory planning forum and to implement periodic performance audits and related activities (Utariningsih, Novalia & Saifullah 2023).

The participatory forum ensures that grassroots aspirations are incorporated into mitigation documents (Ndlovu & Msimanga 2023). At the same time, periodic audits are necessary to prevent the recurrence of operational errors and enhance future management effectiveness (Monsalve et al. 2024). Through this policy framework, it is expected that the OPRB will possess a robust operational foundation that is not contingent upon changes in village leadership.

The second approach involves formalising local wisdom into standard operating procedures (SOPs). The evaluation of the emergency response phase reveals a dualism in social capital: on one hand, citizen initiative serves as a primary logistical strength; yet on the other, the influence of local figures creates challenges in information management because the OPRB has not yet fully established itself as the primary authority. To address this contradiction, the OPRB requires a formal strategy to accommodate local wisdom. This implies that the role of traditional leaders must be recognised as part of a supporting early warning system that operates in parallel with the scientific approach. Conversely, the independent initiatives of residents in fulfilling emergency logistics must be adopted into the OPRB’s official logistics protocols. By incorporating local culture into the formal regulatory framework, the OPRB will acquire stronger social legitimacy, thereby enhancing citizen trust and compliance during emergencies (Depari & Lindell 2023).

Finally, participatory decentralisation through hamlet preparedness teams. Structural issues with the top-down approach, which proved effective during the emergency response but emerged as a weakness in the pre- and post-disaster phases, require correction through a participatory approach. Evaluation findings indicate that the stagnation of participation occurs because risk management is concentrated solely within the core village administration. Therefore, authority must be delegated through the establishment of hamlet-level disaster preparedness teams. This measure decentralises authority to the smallest unit, empowering them to prepare risk-reduction plans, execute independent evacuations and determine post-disaster recovery priorities (Sarabia et al. 2020). This approach is expected to shift residents’ paradigms towards acting on self-awareness, thereby realising a sustainably resilient Krinjing Village.

## Conclusion

This study concludes that disaster management in Krinjing Village exhibits varying performance dynamics across each phase. The OPRB has demonstrated strong adaptive capacity during the emergency response phase, driven by substantial social capital and community-based *gotong royong* initiatives. However, significant opportunities for strengthening exist in the pre-disaster and recovery phases, particularly in participatory planning and evaluation mechanisms that have not yet functioned optimally. To bridge this gap, optimising the OPRB’s role requires structural transformation through three key strategies: (1) policy institutionalisation by integrating risk management into the Village Medium-Term Development Plan (RPJMDes), (2) harmonisation of local wisdom into the organisation’s standard operating procedures and (3) participatory decentralisation through the establishment of hamlet-level preparedness teams. The combination of these three strategies is expected to shift the disaster management paradigm from mere responsiveness to sustainable resilience.

While this study provides detailed insights into local disaster governance, it has certain limitations. It primarily offers an internal evaluation from the perspective of core OPRB administrators, with the institution rather than individual members as the focus of analysis. Future research should broaden its scope to include the perceptions of the wider community, especially service beneficiaries, to achieve a more comprehensive assessment of effectiveness. Additionally, studies that examine individual administrator traits, such as age, gender and tenure, could deepen understanding of how these factors influence institutional dynamics.

Considering the notable findings on social capital and gender during the emergency phase, longitudinal studies or detailed quantitative research linking these social factors would be highly valuable for advancing the field of community-based disaster management.
